# Forensic evaluation of atypical Mongolian spots in dark pigmented skin: navigating the differential diagnosis of potential child abuse

**DOI:** 10.1007/s00414-025-03582-3

**Published:** 2025-08-11

**Authors:** Alberto Amadasi, Larissa Amadasi, Lars Oesterhelweg

**Affiliations:** Institute of Legal Medicine and Forensic Sciences, Charité - Universitätsmedizin Berlin, Turmstrasse 21, 10559 Berlin, Germany

## Abstract

This letter addresses the challenges in differentiating Mongolian spots from traumatic skin lesions in forensic medical evaluations, particularly in cases of suspected child abuse. We appreciate the insightful discussion in the article “Atypical localized Mongolian spots in dark pigmented skin - a challenge for forensic medical examination” which highlights the complexity of diagnosing Mongolian spots, especially when they occur in atypical locations. The case presented here involves a 2-year-old child with unusual skin discolorations on the back and chest, initially suspected to be blunt force injuries. However, upon further investigation and a follow-up examination, these markings were confirmed as atypical Mongolian spots, emphasizing the diagnostic difficulty in distinguishing them from trauma-related bruises. This case underscores the importance of timely and thorough evaluations, including a two-step medico-legal assessment, to prevent misdiagnoses. The letter advocates for increased awareness and education among forensic professionals to accurately differentiate Mongolian spots from signs of abuse, particularly in individuals with dark skin.

Dear Editor,

We are writing with reference to the article “Atypical localized Mongolian spots in dark pigmented skin - a challenge for forensic medical examination” [1], published in the *International Journal of Legal Medicine*. We would like to express our appreciation for the authors’ insightful discussion on the challenges faced in the forensic evaluation of skin lesions in individuals with dark pigmentation, particularly in the context of suspected child abuse. The authors effectively highlight the complexity of diagnosing Mongolian spots, especially when they appear in atypical locations that may be mistaken for signs of physical trauma. As the article rightly points out, Mongolian spots are congenital dermal melanocytoses that typically present as bluish or greyish patches, most commonly located in the lumbosacral region (82.6%) [2, 3]. These spots are generally benign and often resolve in early childhood, and they can be observed in individuals of different ethnicities [4, 5]. However, atypical locations, such as the back, thighs, and even the extremities, pose a significant diagnostic challenge [6]. Their presence in these regions, particularly in individuals with darker skin tones, requires careful consideration to avoid misinterpretation as signs of abuse. This issue is particularly pertinent in forensic medicine, where skin findings can play a crucial role in distinguishing between child abuse and other potential causes of injury.

We would like to present a similar case we have recently dealt with that raised medico-legal issues regarding differential diagnosis in the context of a potential child abuse case, due to the presence of bilateral skin discolorations on the posterior part of the rib cage, which initially raised concerns about possible blunt force trauma.

The Youth Welfare Office received a child protection report from a daycare centre, as caregivers had observed suspected “bruises” on the back of a 2-year-old Nigerian child. Thus, forensic medicine was tasked with conducting a medico-legal examination.

During the forensic medical examination of the child, the following findings were recorded (Fig. 1):

**Fig. 1:**
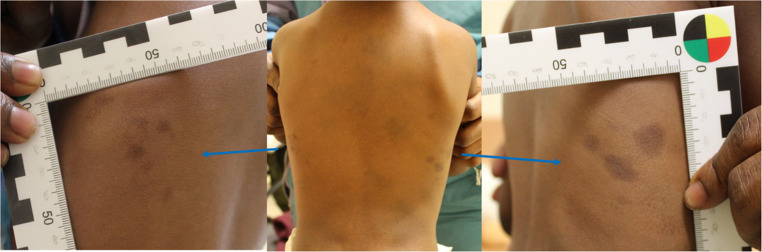
First examination: Presence of lighter spots in the central part of the back, darker and smaller spots on the posterior rib cage (blue arrows)

- Near the spine and on the right side of the upper, middle, and lower back, a total of at least five irregular grey-blue skin discolorations, up to 5 cm in diameter.
- On the right posterior side of the chest, in a 4 cm diameter area, three round, dark brown-violet skin discolorations measuring 0.6 × 0.6 cm, 1.5 × 1.1 cm, and 1.6 × 1.4 cm, respectively. Surrounding them were three patchy, poorly defined, very pale brownish discolorations measuring 0.6 × 0.3 cm, 0.5 × 0.4 cm, and 0.6 × 0.4 cm.
- At approximately the same height on the left posterior side of the chest, in a 4 × 2.5 cm area, five round, irregular dark brown-violet skin discolorations measuring between 0.4 × 0.3 cm and 0.6 × 0.8 cm. Two cm laterally from this area, there was another longitudinal oval, 1.4 × 0.5 cm, pale brownish skin discoloration.

No additional skin alterations or lesions were found during the rest of the examination.

The mother of the child reported that the marks on the back in the median area had been present since birth, whereas the marks, darker in colour and smaller in size, bilaterally at the level of the lower posterior costal arch, were attributed to possible injuries related an argument between the child and a 4-year-old friend, during which the child was pushed and fell to the ground. She reported that the child had fallen “from a chair” or “between two chairs.” The mother further stated that she would never use violence against her child and mentioned ongoing conflicts with the daycare centre, as they sometimes misinterpreted her actions.

The skin discolorations on the upper, middle, and lower right side of the back, from a forensic medical perspective, were actually interpreted as congenital pigment spots in the form of Mongolian spots (congenital dermal melanocytosis) and therefore corroborating the mother’s statements [1–6]. The locally grouped skin discolorations on both posterior sides of the chest, due to their different localisation, shape, and colour, were initially considered compatible with blunt force bruises [7–9]. Regarding the mechanism of injury, the mother’s account (an accidental fall) was deemed incompatible, as the marks were instead considered more likely to result from “grabbing/gripping” injuries, which also aligned with their almost symmetrical appearance at the level of the costal arch. This pattern could potentially be consistent with a two-handed grasping in the chest area. Nevertheless, the simultaneous presence of skin alterations believed to be Mongolian spots did not enable a definitive medico-legal diagnosis.

To ensure a comprehensive evaluation and to determine (or exclude) whether these marks were Mongolian spots with atypical localisation and shape or bruises due to blunt force trauma, the child underwent a second forensic medical examination one month later.

Upon the second examination, all the skin marks on the back appeared unchanged, allowing them to be entirely diagnosed as atypical Mongolian spots (Fig. 2), thereby excluding blunt force injuries and signs of abuse. No further skin alterations were found compared to the examination conducted a month earlier.

**Fig. 2:**
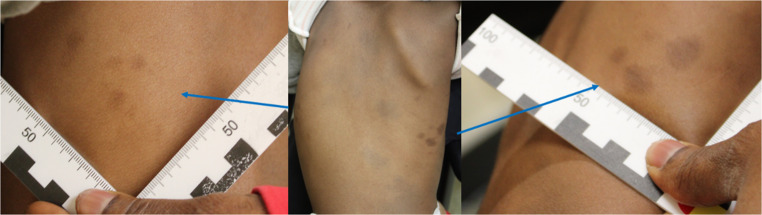
Second examination (one month later): persistence of the same characteristics observed during the first visit, both for the central spots and those on the rib cage (blue arrows). Slight difference in the shape of the most inferior spot on the right due to skin tension at the time of photographic documentation

## Discussion

This case exemplifies the difficulty in differentiating Mongolian spots and bruises in cases of suspected child abuse, particularly when they occur in regions (such as the back) that are typically associated with trauma. In the presented case, the differential diagnosis was particularly complex since the characteristics and localization of the lesions were highly suggestive of “grabbing/gripping” injuries, thus posing a significant risk of misdiagnosis. It is crucial to emphasise that Mongolian spots have been reported in atypical sites such as the back, thighs, and shoulders, sometimes even in combination with more typical locations. As they can indeed present in locations not generally considered typical, this can lead to confusion when evaluating potential child abuse cases [1–5]. A thorough and timely follow-up examination is necessary to distinguish between Mongolian spots and traumatic skin lesions. The persistence of Mongolian spots over time is a key diagnostic feature, as they do not undergo the typical physiological degradation process that characterises bruises and other traumatic injuries. A follow-up examination can provide essential clarity, allowing for the proper classification of skin findings. This is especially important in cases where the lesion’s appearance is ambiguous or atypical. The complexity of this diagnostic process is further compounded by the lack of awareness regarding atypical Mongolian spots. While these lesions are more commonly located in the lumbosacral region, it is vital to consider other areas of the body where they may present. This variability in localisation underscores the critical need for forensic professionals to have a comprehensive understanding of the different patterns of Mongolian spots in order to avoid misdiagnosing them as signs of physical trauma [1, 10, 11].

In conclusion, we would like to emphasise the significance of the findings and the need for continued education on the differential diagnosis of Mongolian spots, particularly in forensic medical examinations. This is essential for forensic professionals to make accurate assessments and avoid the misinterpretation of congenital pigmentation disorders as signs of child abuse. We wholeheartedly agree with the authors’ recommendation [1] for a two-step medico-legal evaluation in cases of skin discolorations in individuals with pigmented skin and the importance of photo documentation for optimal comparability and accurate diagnosis., thus representing a desirable standard in forensic practice in ensuring the identification of abuse while preventing unnecessary and harmful misdiagnoses.

## Data Availability

The Authors confirm that the data supporting the findings of this study are available within the article.
